# Minimizing the Effects of Social Isolation of Horses by Contact with Animals of a Different Species: The Domestic Goat as an Example

**DOI:** 10.3390/ani12172271

**Published:** 2022-09-02

**Authors:** Anna Wiśniewska, Iwona Janczarek, Ewelina Tkaczyk, Izabela Wilk, Wiktoria Janicka, Tomasz Próchniak, Beata Kaczmarek, Elżbieta Pokora, Jarosław Łuszczyński

**Affiliations:** 1 Department of Horse Breeding and Use, Faculty of Animal Breeding and Bioeconomy, University of Life Sciences in Lublin, Akademicka 13 Str, 20-950 Lublin, Poland; 2 Institute of Biological Basis of Animal Production, Faculty of Animal Breeding and Bioeconomy, University of Life Sciences in Lublin, Akademicka 13 Str, 20-950 Lublin, Poland; 3 Department and Clinic of Animal Internal Diseases, Faculty of Veterinary Medicine, University of Life Sciences in Lublin, Akademicka 13 Str, 20-950 Lublin, Poland; 4 Department of Genetics, Animal Breeding and Ethology, Faculty of Animal Science, University of Agriculture in Cracow, 30-059 Cracow, Poland

**Keywords:** horse, isolation, locomotor activity, emotional excitability, goats, paddock, heart rate variability, welfare, social interactions, behavior

## Abstract

**Simple Summary:**

This study examined whether the company of goats in a paddock helps to limit the effects of the social isolation of horses. Four short tests were performed, which examined horses in a herd without goats, horses isolated from the herd without goats, horses in a herd with goats, and horses isolated from the herd with goats. The duration of standing, walking, trotting, and cantering, and the horses’ emotions were determined in each test. The results showed that the company of goats in the paddock only partially limited the effects of the social isolation of horses and reduced their restlessness. However, the horses’ emotions change positively only when goats accompany horses in a herd. Therefore, goats can be used for the planned limiting of movements of isolated horses in paddocks and as an element of environment diversification for horses in a herd. The resulting relaxation helps to reduce the horses’ negative behavior.

**Abstract:**

This study aimed to perform a comparative analysis of the horses’ heart rate parameters and locomotor activity in a herd or isolation, with or without the company of goats. Twenty horses were tested in a paddock, accompanied (or not) by three goats. The experiment comprised four tests (a control test of a herd of horses without goats, a horse isolation test without goats, a test of a herd of horses with goats and a test of an isolated horse with goats). The horse’s locomotor behavior, and the HR, RR, rMSSD, LF, HF, and LF/HF were recorded. The data analysis included a 15-min rest, procedural and recovery HR/HRV periods, and a 5-min period at the beginning of the test. The duration of the horses standing in the company of goats increased significantly. The rMSSD parameter was the significantly lowest in the test of a herd of horses with goats. The company of goats in a paddock does not eliminate the emotional effects of the phenomenon. However, the locomotor behavior decreases. Goats in a paddock can provide a positive distraction for horses in a herd as a decrease in emotional excitability can be regarded as having a relaxing impact on a different animal species.

## 1. Introduction

In both in Poland and in other European countries there is a return to sustainable agriculture [1,2]. In Poland, the activity of ecological farms and agritourism farms using horses as the only workforce plays a significant role in terms of GDP [3]. A similar situation is observed in countries of Western and Southern Europe. It is also worth remembering that horse breeding itself plays a large role in agricultural activity [4]. This breeding is associated with various types of activities carried out by humans. Some of the activities are negative for animals’ welfare, such as the necessity of social isolation [5].

Social isolation has a highly negative impact on gregarious animals [6]. It is known to induce strong stress in many species, which manifests itself in behavioral and physiological changes, e.g., increased vocalization, locomotion, heart rate, or cortisol level [7]. These changes reduce the animal’s utility value and the level of human safety when handling it [8]. In consequence, they weaken the stressed animal and, ultimately, have a crippling effect on it [6,9]. In the case of many methods of horse use, isolation can be reduced the most [10,11]. However, it cannot usually be avoided. The need to isolate horses for multiple reasons results in constantly seeking methods to minimize its effects [10,12,13]. However, one should note that even partial isolation, such as keeping horses in individual boxes, has a negative impact on their welfare [8,14,15]. It does not seem possible to change this, as even a large pasture area is usually an extravagance, and horses grazing in a large group may suffer an injury, which scares their owners for multiple reasons [16,17].

It is necessary to seek new methods to prevent the effects of isolation since the current methods are not fully effective [18,19]. One should note that the social support from the herd is one of the crucial values of gregarious life [20,21]. Social support is usually defined with respect to people [22]. It protects against the health consequences of living under stress, helps to live through a crisis and accelerates the recovery process. A group making up a stable, social unit is found equally often in the animal world [23,24,25]. Horses also form herds with strictly defined social relations [6,26]. Therefore, vicarious social support for individuals without their own herd can be a crucial approach to promoting the physical and mental well-being of farm animals [27]. However, it should be noted that the actual social buffering takes place when the presence of one animal alleviates the stress on another experiencing an unpleasant event and/or subsequently helps the animal to regenerate after it subsides [28].

Since they are not only bred, but also trained, horses often come into relations with humans [29]. These relations take place on multiple levels and concern many disciplines [30,31]. Horses are so sensitive and respond differently to the smell of people that they carry on their clothes and hands, and even to their photographs, the position of their bodies, or the tone of voices [32,33,34,35,36]. They also recognize stress in humans [37]. This is because horses possess a cross-modal ability to recognize individuals based on unique auditory, visual, and olfactory information [38]. Moreover, horses can remember earlier experiences of working with humans or negative relations with them [32]. Therefore, one cannot claim that a person can replace the feeling of the presence of even a micro-herd, although the bond between a horse and a human increases in proportion to the degree of isolation from other animals of the same herd. Examples include relations in unnatural horse herds formed by humans for easier management. Hence, the human’s role in improving the welfare of the horse is crucial.

Therefore, it is worth considering relations between various animal species [39,40]. Companion animals can maintain relations, with cats and dogs being examples of this [41]. Such relations also occur among farm animals, examples of which include lambs and heifers [42], or horses and sheep grazing together [43]. Domestic goats have been suggested by equestrian forums and professional websites as a species which can provide social support to horses during a period of isolation [44,45].

Therefore, a hypothesis was tested that goats decrease horses’ emotional excitability, but only during a period of isolation in a paddock. However, they do not affect horses which are in a paddock in a group. Therefore, the aim of this study was to perform a comparative analysis of the horses’ heart rate parameters and locomotor activity when they were in a herd or in isolation, with or without the company of goats.

## 2. Materials and Methods

The experiment was conducted at the equestrian center at the University of Life Sciences in Lublin, in the east of Poland (51°13′36.93″ N; 22°38′29.85″ E, altitude: 210 m).

### 2.1. Horses

The experiment included 20 clinically healthy, warmblood adult horses (ten geldings and ten mares). The horses had been kept for at least 36 months in one stable. Boxes with straw bedding and a size of 3.5 m × 3.5 m, with grilles in the upper parts of the walls, were situated in two rows separated by a passage corridor. Another corridor separated the stable into two sections, with each one containing two rows of five boxes, with each group of boxes opposite each other. There were geldings kept in one section and mares in the other. The horses were fed three times daily with 9 kg hay, 3 kg of a feeding mixture for recreational horses, and 100 mg of mineral and vitamin concentrate. The feed was given in three equal doses. Water and salt cubes were available ad libitum. The horses had been pastured (grazing period) or paddocked for 4–6 h daily in four groups (groups of boxes) for at least 36 months before the beginning of the experiment.

They were ridden on recreational group trips and sporadic individual riding for six days a week, 1–2 h daily. Individual riding was the only time when horses were separated from the herd. Before the tests, the horses had not had any visual or auditory contact with any other animal except dogs and cats.

### 2.2. Goats

Three two-year-old, clinically healthy, castrated goats with no horns of the Polish white breed were kept loose on straw bedding in one group in a corral with permanent access to a grassy pen. The corral was situated in a separate part of the farm, on a pasture adjacent to the paddock for mares. They had been kept in the same equestrian center as the horses for 18 months, although the horses under study had never had any contact with them. They were fed hay and commercial feed for ruminants. Water and salt cubes were available ad libitum.

### 2.3. Experiment

The horses were assigned to four groups (two groups of mares and two groups of geldings), with five horses of the same sex kept in the same group of boxes in the stable (group of horses). Therefore, those were the same groups in which the horses were usually taken to the paddock. Before the experiment started, a pentagonal corral for goats (approx. m^2^) was made on the trapeze-shaped, earth-and-sand paddock (728 m^2^) known to the horses. During that time, the horses were accustomed to the presence of the corral for goats for three days, 30 min on each day.

**Figure 1:**
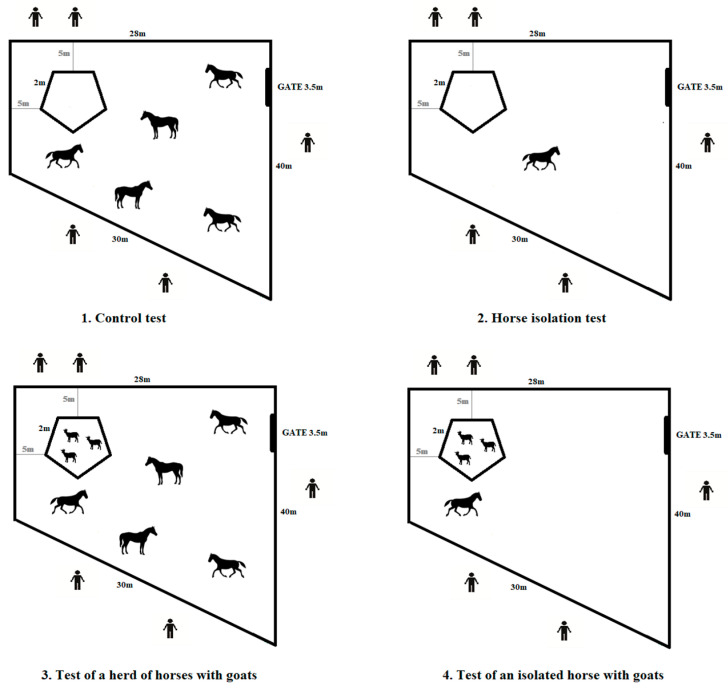
Course of the experiment.

**Figure 2:**
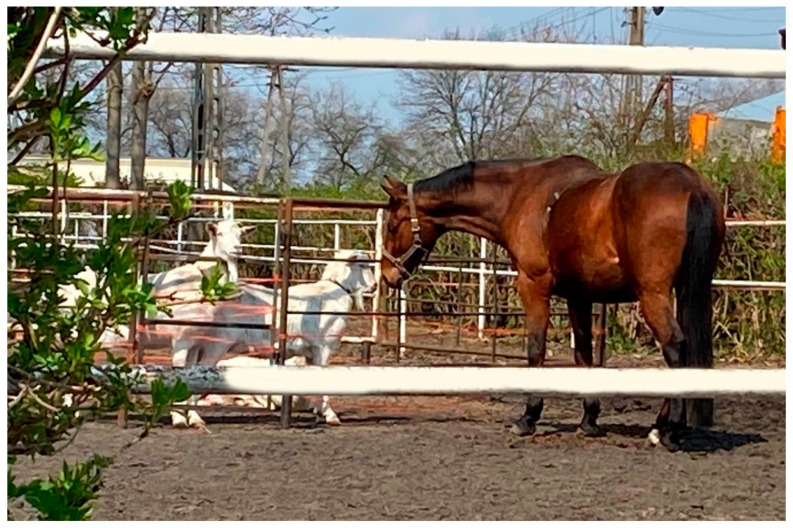
Interactions between a horse and a goat during the experiment.

The experiment comprised four tests of 15 min each (Figure 1 and Figure 2). The first test, called the horse herd control test, involved letting four fixed groups of five animals into a paddock with a corral for goats. Each of the four groups was let out alternately by sex. The second test, called the horse isolation test, started after two days. The horses were let out individually into the same paddock at different times. This period is regarded as sufficient to assess the impact of social isolation on the animals [46]. On the second day after the isolation test, three goats were let out for 60 min into the corridor of the stable where the horses were kept. The horses were accustomed to their presence during that time.

The third test, called the test of a horse herd with goats, was conducted the next day. It involved letting out all the horses in groups into the test paddock with the goats in the corral. The fourth test was called the test of an isolated horse with goats. It involved letting out individual horses in the company of goats in the corral.

All of the tests were started one hour after the morning feeding of the horses. Every effort was made to avoid the horses’ eye contact with other horses or people. However, the usual farm sounds (tractors operating, people talking, cleaning boxes, etc.) could not be avoided. No cases of the horses’ anxiety, becoming interested, or any behavioral reaction to those events were observed.

### 2.4. Locomotor Activity

The horses’ locomotor activity during the tests was recorded by five observers, invisible to the horses. They were able to recognize each animal owing to specific features of their coats and white marks on their heads and legs. The horses’ locomotor activities were recorded by registering all the events [47]. The total duration (s) of walking (slow, four-beat gait), trotting and cantering together (brisk, two- or three-beat gait) and the duration of standing were determined.

### 2.5. Heart Rate and Heart Rate Variability Parameters

The horses’ emotional excitability was determined by analyzing the heart rate (HR) and heart rate variability (HRV). The measurements were performed with a Polar ELECTRO OY, Kempele, Finland, RS800CX type, with an H2 transmitter. The horses were accustomed to the devices. The electrodes were fastened with a strap at the place of a girth, on the left side of the chest, at the heart level. The rubber part of the strap at the place where the electrodes were fixed was covered with large amounts of gel for ECG to optimize conduction and minimize the electric resistance [48]. Subsequently, HR/HRV (heart rate/heart rate variability) monitors, synchronized with specific transmitters, were fixed to elastic straps at the horse’s breastbone level. The data recording began 15 min before the horses went out into the paddock (rest HR/HRV) during the test (procedural HR/HRV15). Additionally, the first five minutes of the test were isolated from the procedural HR/HRV period (procedural HR/HRV5). The recording was completed 15 min after the horses returned to the stable (recovery HR/HRV).

The HR monitoring data were transmitted to the computer via an IrDA USB 2.0 Adapter peripheral and subsequently analyzed in PolarProTrainer 5 (v41.2, Kempele, Finland). Low-power filters were applied to eliminate single artifacts. The following parameters were analyzed–HR (beats per minute)–heart rate,–RR interval (ms)–intervals between successive R waves in the QRS complex, rMSSD (ms)–root mean square of successive differences between consecutive RR intervals: time analysis parameter, HF (ms^2^)–high–frequency spectrum power component (0.15–0.4 Hz), LF (ms^2^)–low–frequency spectrum power component (0.04–0.15 Hz), LF/HF (%)–low–frequency spectrum power to high-frequency spectrum power ratio, indicating the sympathetic/parasympathetic balance in ANS (Autonomic Nervous System) [49]. Pursuant to the recommendations of the Task Force of the European Society of Cardiology and the North American Society of Pacing and Electrophysiology [50], the duration of each measurement for short-term analyses was identical. It lasted 15 min, including rest HR/HRV, procedural HR/HRV15, and recovery HR/HRV.

### 2.6. Statistical Methods

The statistical analyses were performed with a commercial analytical software package–SAS 9.4 (SAS Institute Inc., Cary, NC, USA) [51].

The feature distribution analysis was based on the Kolmogorov–Smirnov, Cramer-von Mises, and Anderson–Darling tests at α = 0.05. The main descriptive statistics for the features were presented: number of observations, M—arithmetic mean, SE—standard error of the mean, SD—standard deviation, V—coefficient of variance, Me—median, Min—minimal and Max—maximum value of an observation.

The significance of the impact of constant factors on the features under study was verified by the multifactorial analysis of variance (GLM procedure) with the following model:

Y_ijklm_ = μ + d_i_ + p_j_ + w_k_ + e_ijklm_

where: Y is the feature under analysis, μ the mean for the feature, d theconstant impact of the test day, p the constant impact of the animal’s sex, w the constant impact of the horse’s age, and e the remainder, unexplained by the experiment (error).

The significance of the differences between the means was determined with Tukey’s multiple comparison test. The final results are presented as means with standard deviations of the means (SD).

## 3. Results

Table 1 presents the main descriptive statistics for each feature under analysis.

For nearly 30% of the features under study, a significant impact of the subsequent test factor was present (Table 2). This group included all of the three locomotor features, three mean values of the parameters from the procedural15 period (HR—heart rate, rMSSD—root mean square of successive differences between consecutive RR intervals, LF/HF—low-frequency spectrum power to high-frequency spectrum power ratio), three from the recovery period (HR—heart rate, rMSSD—root mean square of successive differences between consecutive RR intervals, LF–low–frequency spectrum power) and one parameter from the rest period (LF/HF–low–frequency spectrum power to high-frequency spectrum power ratio).

The duration of standing in the paddock was the significantly longest in the test of horse herd with goats and the test of an isolated horse with goats (Table 3). This feature had the lowest value in the horse isolation test. The duration of standing in the control test had an average value. The duration of walking was significantly longer than the others during the horse herd control test, and the shortest was in the horse isolation test. The values were average in the other two tests. The trotting/cantering duration was significantly longer than the others during the horse isolation test. The differences in the other features were not significant.

The resting HR did not differ significantly during consecutive tests (Table 4). The procedural HR5 was significantly higher than the others in the horse isolation test and in the test of isolated horses with goats. The procedural HR15 was the highest in the test of isolated horses with goats. The value was similar to that in the horse isolation test. The lowest procedural HR15 was observed in the test of a horse herd with goats. It was similar to that in the horse herd control test. The values in tests I and II were also similar. The recovery HR was the highest in the test of an isolated horse with goats and in the horse isolation test. The latter value was also similar to the value observed in the horse herd control test and in the test of a horse herd with goats.

There were no significant differences between the rest RR in consecutive tests (Table 5). The procedural RR5 was significantly higher than the others in the horse herd control test and in the horse herd with goats test. The procedural RR15 was significantly higher than the others in the horse herd control test and in the test of horse herd with goats. The latter value was also similar to that observed during the isolated horse test. Furthermore, it was similar to the value in the test of an isolated horse with goats.

The rest rMSSD and procedural rMSSD5 did not differ significantly during consecutive tests (Table 6). For the procedural rMSSD15 and recovery rMSSD, values higher than the others were observed in the horse herd with goats test and in the horse herd control test, with the latter value also similar to those found in the isolated horse test and in the test of an isolated horse with goats.

The remaining LF, procedural LF5 and LF15 did not differ significantly during consecutive tests (Table 7). Significant differences were observed for the recovery LF. The value for the test of horse herd with goats was the highest, and value for the test of an isolated horse with goats was the lowest. The values of this parameter were close to the highest and the lowest values in the other two tests.

The LF/HF value for the test of a horse herd with goats and one for the test of an isolated horse with goats was the highest (Table 8). For the latter test, this parameter was also similar to the one in the horse herd control test and in the isolated horse test.

Table 9 shows the features significantly affected by the sex factor. The duration of a mare standing during the tests was significantly shorter than a gelding standing (Table 10). The duration of walking proved to be significantly longer in the mares compared with the geldings. The recovery HR (heart rate) in the mares was significantly higher than in the geldings. It was the opposite in the case of the recovery RR (RR interval).

## 4. Discussion

The experimental design applied in the study significantly diversified the locomotor activity of the horses. The presence of goats in the paddock prolonged the standing duration of horses, regardless of whether the horses were in a herd or isolated. This time was the shortest when horses were isolated without the company of goats. The walking duration was the shortest when the horses were isolated without the company of goats. As expected, the trotting and cantering duration was then much longer than in the other situations. Interestingly, horses in a herd with or without goats, or isolated horses with goats, walked, trotted, or cantered during a comparable time. On the other hand, no impact of the goats on the locomotor activity of horses in a herd was observed. This may mean that the presence of other animals of the same species is essential. Therefore, it could be hypothesized that the company of goats can reduce horses’ locomotor activity, but only during their isolation. However, it seems that these findings can be seen as satisfactory in minimizing the effects of social isolation of horses. Krueger et al. [52] report that social isolation is disadvantageous to gregarious animals. Specific behavior then becomes more intensive, such behavior including increased locomotor activity, among many features. Let it be emphasized at this stage of research that an analysis of the locomotor features provides grounds for suggesting a positive impact of the goat company on minimizing the effects of horse social isolation.

However, it seems interesting how the experiment proposed in this study affected the horses’ emotional excitability. Social isolation triggers negative emotions, which manifest themselves mainly by an HR (heart rate) increase and a decrease in the parameters indicative of parasympathetic activity of the ANS (Autonomic Nervous System) [53]. These findings proved to be partially different from those presented for the locomotor features. The difference is visible for the HR (heart rate) and RR (intervals between successive R waves in the QRS complex). Social isolation caused unwanted changes in these parameters regardless of whether the company of goats was present or not. This was the most manifest during the first five minutes of the horses’ stay in the paddock. The parameters from 15 min of the test and from the recovery period were not so unambiguous, although they confirmed the differences present during the five minutes. Therefore, it may be suggested that the animals experience strong emotions during the first moments of isolation, which is confirmed by the findings of studies conducted by Moons et al. [54] and Mal et al. [55]. The horses’ emotional excitability is stabilized during subsequent minutes, although the stabilization is not significant enough to bring the organism to the resting state during the 15 min of the recovery period. Therefore, it can be claimed that the company of goats does not block the growth of the emotional excitability in isolated horses. Perhaps a longer time spent with goats by the isolated horses than that used in the study would have given different results. However, our research initially focused on the phenomenon of short-term social isolation. Interestingly, the goats’ company calms horses in a herd. Horses probably perceive those animals as a type of distraction from the pasture behavior. Patkowski et al. [56] are of a similar opinion.

Regarding the study hypothesis, it can be partially corroborated at this stage. The company of goats certainly limits the locomotor activity of the isolated horses, although, unfortunately, it does not decrease the emotional excitability, which is a consequence of isolation. The current findings are consistent with those published by other authors, who point out the discrepancies between the situational behavior demonstrated by horses and the emotions experienced at the time [57,58]. Moreover, according to Lenoir et al. [59] and Rietmann et al. [60], HR (heart rate) and RR (intervals between successive R waves in the QRS complex) are sometimes difficult to interpret due to factors other than emotional factors, which can change the values under study. In contrast, the parameters which clearly show the activity of both components of the autonomic nervous system are beneficial in this case [49]. Not all the HRV (heart rate variability) parameters may have been significantly different with respect to the subsequent test factor, but the findings allow for a sufficiently detailed analysis of the changes in this regard. First of all, the factor in question diversified the rMSSD (root mean square of successive differences between consecutive RR intervals), i.e., the parameter indicating the activity of the parasympathetic part of the ANS (Autonomic Nervous System), and also LF (low-frequency spectrum power), which indicates the activity of the sympathetic part of the ANS [53]. This group also included LF/HF (low-frequency spectrum power to high-frequency spectrum power ratio) as information on the balance of the ANS [61]. The sympathetic system activity increases considerably under stress, and it is associated with adrenaline and noradrenalin secretion, which accelerate the heart rate. Acetylcholine, whose level grows with increasing activity of the parasympathetic nervous system, has the opposite action [62]. Therefore, hormonal reactions are accompanied by an increase in the sympathetic system’s advantage over the parasympathetic system, which manifests itself as an increase in the LF/HF ratio. 

Therefore, the group of HRV (heart rate variability) parameters examined in this study can be regarded as sufficient to determine the horses’ emotional excitability in different variants of this experiment. At the start of discussing those parameters from rMSSD (root mean square of successive differences between consecutive RR intervals), one should emphasize that significant differences were present only within the procedural and recovery periods. It is manifest that the horse isolation, regardless of whether it was with or without the company of goats, reduced the sympathetic activity of the ANS (Autonomic Nervous System) considerably. Therefore, one cannot confirm the positive impact of goats on horses’ emotions during the latter’s isolation, which should be regarded as an extremely disadvantageous phenomenon in horses’ lives. This finding has been corroborated by other authors [63]. The current study found similar results in the LF/HF (low-frequency spectrum power to high-frequency spectrum power ratio), but only for the whole test. It is notable that the company of goats had a noticeable, positive impact on the emotions of horses staying in a herd in the paddock. The rMSSD had the highest values during the test and after it was completed, which may be indicative of the considerable relaxation of the horses. Although the findings may not corroborate the hypothesis proposed in this study, they shed new light on the positive impact on the horses’ emotions. Introducing new animal species into a herd of horses can alleviate bad emotions, tension, and hierarchical disturbances.

Therefore, social support provided by goats to animals in a herd formed by humans to make its management easier is possible. Problems related to the isolation of horses are not solved in an emotional sphere, although the effect is noticeable from the behavioral point of view. The results may be interpreted in this manner, although they are not fully confirmed for LF (low-frequency spectrum power). First, the differences appeared only during the recovery period. Second, the highest value was observed (paradoxically) during the test of the herd with goats, and the lowest value was observed during the test of an isolated horse with goats. This result is therefore confirmed by those concerning locomotor features. At the same time, it is completely inconsistent with the other heart rate parameters when it is analyzed as a determinant of excitation from the sympathetic part of the ANS (Autonomic Nervous System) [64]. However, when this parameter is analyzed with respect to the excitation both of the sympathetic and parasympathetic systems [65], the results can be regarded as largely consistent with those concerning the other parameters.

## 5. Conclusions

The company of goats in a paddock does not provide full social support for isolated horses, as it does not eliminate the emotional effects of the phenomenon. The locomotor behavior decreases, which may contribute to planned restriction of horse movements when they have to be kept in a paddock individually. Goats in a corral in a paddock can provide a positive distraction for horses in a herd. A decrease in emotional excitability, which is then observed, can be regarded as a relaxing impact on a different animal species.

## Figures and Tables

**Table 1:** Main descriptive statistics for the features under analysis.

Variable	N	M	SE	SD	V	Me	Min	Max
Standing (s)	80	478.16	15.07	134.80	18,169.88	28.19	483.50	94.00
Walk (s)	80	353.34	12.18	108.92	11,864.15	30.83	351.50	129.00
Trotting/cantering (s)	80	68.50	11.64	104.09	10,833.92	151.95	14.50	0.00
Rest HR (b.p.m.)	80	37.25	0.33	2.93	8.57	7.86	38.00	30.00
ProceduralHR5 (b.p.m.)	80	63.59	2.52	22.52	506.95	35.41	56.50	37.00
Procedural HR15 (b.p.m.)	80	62.44	2.29	20.47	419.10	32.79	55.00	37.00
Recovery HR (b.p.m.)	80	55.21	2.01	17.94	321.92	32.50	51.00	34.00
Rest RR (ms)	80	1619.11	14.23	127.27	16,198.05	7.86	1594.00	1265.00
ProceduralRR5 (ms)	80	1038.00	32.69	292.42	85,511.47	28.17	1041.50	399.00
Procedural RR15 (ms)	80	1057.84	31.57	282.38	79,738.40	26.69	1076.00	476.33
Recovery RR (ms)	80	1182.70	35.28	315.53	99,556.16	26.68	1181.00	507.00
Rest rMSSD (ms)	80	106.15	3.24	29.00	840.71	27.32	101.65	10.90
Procedural rMSSD5 (ms)	80	77.58	5.51	49.32	2432.73	63.58	67.25	4.30
Procedural rMSSD15 (ms)	80	73.99	4.55	40.74	1659.42	55.06	68.37	8.87
Recovery rMSSD (ms)	80	74.97	4.92	44.02	1937.93	58.72	73.15	13.50
Rest LF (ms^2^)	80	3931.74	237.03	2120.10	4,494,815.52	53.92	3533.68	51.15
Procedural LF5 (ms^2^)	80	4019.45	391.93	3505.56	12,288,924.73	87.21	2934.98	43.84
Procedural LF15 (ms^2^)	80	3895.49	322.25	2882.33	8,307,812.98	73.99	3046.94	225.38
Recovery LF (ms^2^)	80	3612.34	347.24	3105.77	9,645,799.19	85.98	2906.99	103.90
Rest HF (ms^2^)	80	2708.28	179.55	1605.92	2,578,976.32	59.30	2194.92	20.70
ProceduralHF5 (ms^2^)	80	2361.73	388.50	3474.85	12,074,564.67	147.13	1108.59	9.16
Procedural HF15 (ms^2^)	80	1972.72	244.08	2183.12	4,765,997.69	110.67	1226.81	23.89
Recovery HF (ms^2^)	80	2256.28	384.77	3441.51	11,844,020.80	152.53	1354.54	33.52
Rest LF/HF (%)	80	148.26	6.57	58.76	3452.34	39.63	148.00	37.30
Procedural LF/HF5 (%)	80	338.68	32.03	286.50	82,080.08	84.59	243.00	36.00
Procedural LF/HF15 (%)	80	365.57	26.44	236.50	55,930.83	64.69	284.68	35.50
Recovery LF/HF (%)	80	359.33	56.37	504.15	254,166.12	140.30	230.05	22.20

**Table 2:** The features for which a significant impact of the subsequent test factor was observed (*p* ≤ max 0.05).

Feature	DF	Square Sum Type 3	Mean Square	Value of F	Pr. > F
Standing	3	328,262.2375	109,420.7458	7.69	0.0002
Walking	3	139,415.7375	46,471.9125	4.56	0.0055
Trotting/cantering	3	571,903.3000	190,634.4333	51.74	<0.0001
Procedural HR15	3	9007.261111	3002.420370	9.69	<0.0001
Recovery HR	3	4784.437500	1594.812500	6.12	0.0009
Procedural rMSSD15	3	14,621.69028	4873.89676	3.22	0.0276
Recovery rMSSD	3	27,211.62250	9070.54083	5.66	0.0015
Recovery LF	3	87,147,953.37	29,049,317.79	3.33	0.0241
Rest LF/HF	3	41,438.38037	13,812.79346	4.65	0.0050
Procedural LF/HF15	3	633,809.6458	211,269.8819	4.21	0.0083

**Table 3:** Locomotor features in consecutive tests.

Feature	Standing		Walking		Trotting/Cantering	
Test	Mean	SD	Mean	SD	Mean	SD
I	458.35 AB	82.04	419.15 A	81.21	22.50 B	20.22
II	381.90 B	144.63	303.20 B	108.79	214.90 A	111.62
III	533.25 A	149.12	346.70 AB	127.20	20.05 B	35.13
IV	539.15 A	91.59	344.30 AB	85.92	16.55 B	29.06

The means marked with different capital letters (A, B) are significantly different at α = 0.01. I: control test of a horse herd, II: horse isolation test, III: test of a horse herd with goats, IV: test of isolated horse with goats.

**Table 4:** HR (heart rate) during consecutive tests.

Parameter	Rest HR		Procedural HR5		Procedural HR15		Recovery HR	
Test	Mean	SD	Mean	SD	Mean	SD	Mean	SD
I	36.00	2.05	55.70 B	14.61	56.00 BC	9.82	49.85 B	9.04
II	37.70	2.36	72.50 A	25.95	69.83 AB	22.84	58.80 AB	19.82
III	37.15	2.50	49.65 B	4.61	48.67 C	4.96	46.20 B	6.35
IV	38.15	4.11	76.50 A	25.90	75.27 A	25.02	66.00 A	23.91

The means marked with different capital letters (A, B) are significantly different at α = 0.01. I: control test of a horse herd, II: horse isolation test, III: test of a horse herd with goats, IV: test of isolated horse with goats.

**Table 5:** RR (RR interval) during consecutive tests.

Parameter	Rest RR		Procedural RR5		Procedural RR15		Recovery RR	
Test	Mean	SD	Mean	SD	Mean	SD	Mean	SD
I	1670.40	101.20	1142.30 A	277.12	1121.87 AB	194.20	1241.85 AB	225.07
II	1595.60	96.80	928.15 B	310.91	959.80 BC	312.87	1128.65 B	361.71
III	1620.55	121.98	1215.15 A	132.99	1251.50 A	144.96	1323.75 A	182.03
IV	1589.90	169.14	866.40 B	276.07	898.20 C	306.90	1036.55 B	386.18

The means marked with different capital letters (A, B) are significantly different at α = 0.01. I: control test of a horse herd, II: horse isolation test, III: test of a horse herd with goats, IV: test of an isolated horse with goats.

**Table 6:** rMSSD (root mean square of successive differences between consecutive RR intervals) during consecutive tests.

Parameter	Rest rMSSD		Procedural rMSSD5		Procedural rMSSD15		Recovery rMSSD	
Test	Mean	SD	Mean	SD	Mean	SD	Mean	SD
I	97.74	23.71	79.19	33.08	73.14 AB	29.74	77.43 AB	35.01
II	105.01	22.46	65.66	51.04	62.89 B	42.14	63.78 B	42.10
III	114.92	38.09	92.51	37.71	96.33 A	34.76	103.75 A	42.46
IV	106.95	28.64	72.96	67.71	63.61 B	47.55	54.94 B	42.71

The means marked with different capital letters (A, B) are significantly different at α = 0.01. I: control test of a horse herd, II: horse isolation test, III: test of a horse herd with goats, IV: test of an isolated horse with goats.

**Table 7:** LF (low-frequency spectrum power) during consecutive tests.

Parameter	Rest LF		Procedural LF5		Procedural LF15		Recovery LF	
Test	Mean	SD	Mean	SD	Mean	SD	Mean	SD
I	3142.55	1919.71	3778.31	3144.74	3928.05	3131.71	3494.14 AB	3453.20
II	3982.10	2053.94	3877.92	3177.72	3664.58	2627.37	4041.42 AB	3644.05
III	4721.39	2313.17	4746.33	3727.34	4769.70	2791.97	4890.61 A	2445.00
IV	3880.90	2029.02	3675.25	4056.18	3219.63	2948.68	2023.21 B	2082.74

The means marked with different capital letters (A, B) are significantly different at α = 0.01. I: control test of a horse herd, II: horse isolation test, III: test of a horse herd with goats, IV: test of an isolated horse with goats.

**Table 8:** LF/HF (low-frequency spectrum power to high-frequency spectrum power ratio) during consecutive tests.

Parameter	Rest LF/HF		Procedural LF/HF5		Procedural LF/HF15		Recovery LF/HF	
Test	Mean	SD	Mean	SD	Mean	SD	Mean	SD
I	125.74 B	51.96	276.89	171.72	346.85 AB	212.12	274.80	142.77
II	136.66 B	43.01	432.75	400.82	449.82 A	304.44	525.83	882.33
III	185.91 A	62.51	243.48	137.85	227.70 B	68.58	203.28	97.74
IV	144.73 AB	60.96	401.61	324.17	437.92 A	238.35	433.40	424.55

The means marked with different capital letters (A, B) are significantly different at α = 0.01. I: control test of a horse herd, II: horse isolation test, III: test of a horse herd with goats, IV: test of an isolated horse with goats.

**Table 9:** The features found to be significantly affected by the sex factor (*p* ≤ 0.1).

	DF	Square Sum Type 3	Mean Square	Value of F	Pr. > F
Standing	1	54,123.50	54,123.50	3.80	0.0550
Walking	1	34,804.05	34,804.05	3.42	0.0686
Recovery HR	1	743.77	743.77	2.85	0.0955
Recovery RR	1	319,594.41	319,594.41	3.74	0.0570

**Table 10:** Significantly differing features within the sex factor.

Feature	Standing		Walking		Recovery HR		Recovery RR	
Sex	Mean	SD	Mean	SD	Mean	SD	Mean	SD
Mares	449.45 b	124.17	377.83 a	95.54	59.28 a	19.69	1108.28 b	313.22
Geldings	506.88 a	140.34	328.85 b	116.94	51.15 b	15.19	1257.13 a	303.63

The means marked with different lowercase (a, b) are significantly different at α = 0.05.

## Data Availability

The data presented in this study are available on request from the corresponding author.
